# New Frontiers in Retina: highlights of the 2020 angiogenesis, exudation and degeneration symposium

**DOI:** 10.1186/s40942-020-00221-4

**Published:** 2020-05-22

**Authors:** Carmen A. Puliafito, Charles C. Wykoff

**Affiliations:** 1 Retinal Synthesis LLC, Pasadena, CA USA; 2 Houston Methodist Hospital, Weill Cornell Medical School, Houston, TX USA

**Keywords:** Geographic atrophy, Neovascular AMD, Diabetic retinopathy, Imaging, Artificial intelligen

## Abstract

We summarize the most important findings presented at the 2020 angiogenesis, exudation and degeneration symposium in five topic areas: (1) epidemiology of retinal vascular disease and macular degeneration; (2) dry AMD and geographic atrophy; (3) neovascular age-related macular degeneration; (4) drug delivery and devices and (5) diabetic retinopathy.

## Background

The *Angiogenesis, Exudation, and Degeneration Symposium* has proven to be one of the most important meetings for the retina space [1]. The 16th annual meeting, sponsored by the Bascom Palmer Eye Institute, was held in Miami, Florida on February 8th, 2020. The program provides a comprehensive review of key new developments in retinal pharmacotherapy, including updates on new drugs and delivery systems aimed at improving treatment options for the early stages of age-related macular degeneration (AMD), the advanced stages of geographic atrophy (GA) and neovascular AMD (nAMD), and the many manifestations of diabetic retinopathy (DR) including diabetic macular edema (DME), proliferative DR (PDR) and non-proliferative DR (NPDR). Together, AMD and DR remain two of the greatest causes of visual disability in developed nations (2). Advances in imaging and optimization of artificial intelligence algorithms for guiding prognosis and management of both AMD and retinal vascular disease were also discussed and actively debated [2].

Within a framework of the major retinal diseases, the current manuscript highlights what we believe to be the most insightful, relevant and important topics for the vitreoretinal community to recognize at the dawn of a new decade.

## Epidemiology of retinal vascular diseases and macular degeneration

Dr. Andrew Moshfeghi reported on the epidemiology of the diseases managed in retinal practices across the United States from 2014 to 2019 by evaluating the electronic medical records of more than 300 retinal specialists. Eyes with either a retinal vascular disease or AMD accounted for more than half of all eyes evaluated and the prevalence of both increased across the 6-year period. The prevalence of specific retinal diseases included 16% with the early, intermediate or late stages of dry AMD; 13% with neovascular AMD (nAMD); 10% with diabetic macular edema (DME); 8% with diabetic retinopathy without DME; 3% with branch retinal vein occlusion (RVO); 2% with central RVO.

## Dry AMD and geographic atrophy

The dry forms of AMD, in particular GA, remain the largest unmet need in retinal disease management [3] and many of the ongoing clinical trial programs, including 2 of which are currently being investigated in global phase 3 programs, were described in detail. Two study programs focused on earlier stages of dry AMD and highlighted the under-appreciated need for considering the visual dysfunction such patients experience.

Dr. Scott Cousins provided an update of the ReCLAIM Trial which investigated the use for elamipretide (Stealth BioTherapeutics, Cayman Islands) for the treatment of vision loss associated with intermediate dry AMD and noncentral GA. Elamipretide, a mitochondrial protective drug, is hypothesized to improve vision and dark adaption in dry AMD. Eyes with either noncentral GA or high risk drusen with BCVA ≥ 55 letters and low luminance deficit were treated with 40 mg of elamipretide subcutaneously once daily for 24 weeks. Exploratory endpoints included change in BCVA, low luminance visual acuity (LLVA) and low luminance reading acuity (LLRA). In the drusen cohort, BCVA improved 3.6 ± 6.4 letters (p = 0.025) and LLVA improved 5.6 ± 7.8 letters (p = 0.006). In the noncentral GA cohort, GA area growth was reduced 50% compared to historical controls. Dark adaption (DA) is a difficult outcome to quantify due to variability in disease severity, patient effort and methodology. In spite of these limitations, results were suggestive elamipretide therapy can improve DA in some subjects. In the drusen cohort dark adaption improved at 2 or more visits in 47% of eyes and in the noncentral GA cohort in 38% of eyes. Elamipretide continues to be studied in ongoing human clinical trials [4].

Dr. Glenn Jaffe and Dr. Peter Kaiser presented results from a Phase 2 trial of risuteganib (Luminate, Allegro Ophthalmics, San Juan Capistrano, CA). The study suggested that some of the structural changes observed in dry AMD may be able to be ameliorated with an associated improvement in visual function [5]. Risuteganib is a synthetic oligopeptide purported to regulate select integrin functions involved in the pathogenesis of dry AMD. In preclinical studies, risuteganib protected retina cells against cytotoxins such as peroxide. Forty-five patients with a wide range of phenotypes of dry AMD and best corrected VA (BCVA) between 20/40 and 20/200 were treated with either intravitreal 1.0 mg risuteganib or sham injection. A key inclusion criteria was symptomatic decrease in VA over the past year. At week 16, patients in the risuteganib group received a second 1.0 mg risuteganib dose. Outcomes from the active treatment arm at week 28 were compared to outcomes from the control arm at week 12, an unusual study design. A gain of ≥ 8 letters from baseline was observed in 48% of active treated patients at week 28 vs 7% of sham patients at week 12. A gain of gained ≥ 15 letters was observed in 20% of active treated patients at week 28 vs no sham patients at week 12. By OCT analysis, greater outer retinal and photoreceptor thickness and volume and smaller ellipsoid zone defects within the central 1 mm zone at baseline were associated with increased BCVA response to risuteganib. A larger, randomized, masked study (estimated to be approximately 345 patients) is planned to study the proportion of patients gaining ≥ 15 letter gain as the primary endpoint.

Dr. Cedric Francois presented new observations regarding the pathogenesis of exudative AMD (E-AMD) noted in 11% of eyes in the Phase 2 FILLY trial of pegcetacoplan (APL-2; Apellis, Crestwood, KY), an inhibitor of C3 cleavage [6]. APL-2 appears to be one of the most promising agents for treatment of GA and is being studied in a global phase 3 program involving an estimated 1200 patients [7]. The hypothesis described is that C3 breakdown products including C3b can overload the retinal pigment epithelium (RPE) causing an energy deficit leading to cellular dysfunction and subsequent photoreceptor injury and death. In the Phase 2 trial involving 246 subjects, eyes were treated with intravitreal injections of 15 mg/0.1 ml of APL-2 monthly (M) or every other month (EOM) for 12 months, with GA growth being reduced by 20% and 29% in the EOM and M groups respectively. One unexpected finding from the FILLY trial was the development of investigator-determined E-AMD occurred in 9% and 21% of eyes in EOM and M groups respectively. History of nAMD in the contralateral eye at baseline was a risk factor for development of study eye E-AMD. Specifically, 69% of eyes that developed E-AMD had a history of fellow eye nAMD compared to 33% of eyes not developing E-AMD. Furthermore, retrospective review of baseline OCT data found that 73% of eyes that developed E-AMD had the “double layer sign”, compared to 32% of treated eyes that did not develop. The “double layer sign” has been observed to be associated with quiescent type 1 CNV [8]. The authors speculated that APL-2 may increases exudation from preexisting type 1 CNV. This observation suggests that pretreatment OCT angiography to identify type 1 CNV might be useful in identifying eyes at greater risk for developing E-AMD and thus might benefit from closer clinical monitoring. Importantly, with prompt anti-VEGF treatment, VA did not appear to be negatively affected among eyes developing E-AMD compared to eyes not developing E-AMD.

Dr. Carl Csaky reported data from a pivotal clinical trial of avacincaptad pegol (Zimura, IVERIC bio, New York, NY), an aptamer that inhibits C5 cleavage, for the treatment GA [9]. Prevention of C5 cleavage is projected to prevent inflammasome activation through C5a and prevent membrane attack complex (MAC) formation through C5b. A total of 286 subjects were treated with monthly intravitreal injection of avacincaptad pegol (2 mg and 4 mg study arms) or sham injection [10]. The primary efficacy endpoint was the mean rate of change of GA over 12 months measured by fundus autofluorescence at three time points: baseline, month 6 and month 12 using a square root transformation of GA area to account for differences in growth rates due to difference in baseline lesion size, a well-accepted approach also used in FILLY. The primary efficacy endpoint was achieved for both the 2 mg and 4 mg doses, leading to approximately a 27% reduction in GA growth through 12 months. Development of nAMD was reported in 2.7% of sham eyes compared to 9-10% of the avacincaptad treated patients. A Phase 3 trial studying the 2 mg dose is anticipated.

Dr. Ryan Rich reported results of two Phase 2 studies using a sustained release brimonidine implant (Brimo DDS, Allergan, Irvine, CA) for treatment of eyes with GA. Preclinical studies involving RPE and Muller cells have demonstrated cytoprotective effects with bromonidine. The first trial investigated Brimonidine doses of 132 and 264 Å µg compared to sham among 113 patients; at the 12-month time point, the brimonidine treated eyes demonstrated reduced GA growth by 19% and 28% in the 132 and 264 brimonidine arms compared to sham. In a second trial involving a 400 Å µg implant [11], the GA growth rate was reported to be reduced by 7% and 11% at the 24- and 30-month time points compared to sham. Overall the beneficial effect of brimonidine was more robust among eyes with larger areas of GA at baseline and there was no indication of increased nAMD development in the active treatment groups. The possibility of moving forward into a phase 3 program was discussed.

Dr. Vrinda Hershberger reported results from a Phase 1 trial of GR39821 among patients with GA (Genentech, South San Francisco, CA), a single ascending, multiple-dose study evaluating the safety and tolerability of an intravitreally delivered therapeutic designed to inhibit anti-High Temperature Requirement A1 (HtrA1) [12]. Genome wide association studies have identified *ARMS2/HTRA1* to be a significant locus impacting AMD risk [13]. HtrA1 is a secreted protease expressed by RPE and horizontal cells that degrades extracellular matrix proteins as well as proteins involved in the visual cycle. Inhibition of HtrA1 is hypothesized to slow the progression of GA lesion growth. The Phase 1 study found that intravitreal anti-HtrA1 was well tolerated at doses up to 20 mg/eye, with no dose-limiting toxicities, ocular serious adverse events (AEs), or systemic or ocular AEs related to the anti-HtrA1 pharmaceutical. Furthermore, aqueous humor analyses suggested on target activity of the agent and suggested duration of effect greater than 8-weeks with the higher doses. A phase 2 study of is enrolling.

OCT-A continues to grow as a research tool and is now well-accepted as an important clinical tool in the evaluation of eyes with AMD. Imaging of the choriocapillaris in attempt to understand the role of vascular perfusion in the pathogenesis of AMD is a topic of intense investigation. Dr. Ricky Wang performed volumetric imaging of the choroidal vasculature using swept source (SS) OCT-A and reported that choroidal volume varies widely in normal subjects, the choroid thins with increasing age, and that choroidal vessel density appears independent of age in normal subjects. Dr. Philip Rosenfeld is exploring the role of the choriocapillaris (CC) in AMD progression. Challenging this field, however, drusen cast shadows on the underlying CC and these can interfere with CC flow measurements by OCT-A [14]. In this study, a novel compensation strategy to overcome the effect of these drusen-associated shadows was developed and the strategy was validated by imaging eyes with drusen and shadows and then again after drusen spontaneously collapsed without development of GA, so that no shadows remained.

Dr. Emily Chew discussed the development of a deep learning algorithm to detect features of AMD including drusen, reticular pseudodrusen (RPD), pigmentary changes as well as both forms of late AMD. DeepSeeNet [15] is an artificial intelligence (AI) system designed to assign eyes to a specific AREDS severity scale based on evaluation of color fundus photographs of drusen (by size, small, medium or large) and presence of pigmentary changes, two validated biomarkers for the risk of progression to late AMD at a patient level. This system performed better than clinicians in detecting drusen and pigmentary changes but was inferior to clinicians in the detection of late AMD. Larger training sets may be able to improve DeepSeeNet performance.

## Neovascular age-related macular degeneration

Most exudative retinal diseases are chronic and relapsing, and while treatable with anti-vascular endothelial growth factor (VEGF) medications, they require repeated therapeutic interventions over years, often indefinitely. It has been estimated that in 2019, a total of 24.4 million intravitreal injections were administered globally, with 6.9 million of these injections performed in the United States alone. Developing drugs, devices, and strategies that can reduce this tremendous treatment burden are a priority. Promising approaches include (a) new anti-VEGF-A monotherapies with extended duration of intraocular biological activity, (b) combination approaches targeting cytokines and relevant molecular pathways beyond VEGF-A. (c) novel delivery systems, and (d) gene therapy with the goal of establishing an intra-ocular bio-factory to produce an anti-VEGF protein continuously.

Dr Diana Do presented an update related to an ongoing, open label, Phase 1b study of a next-generation intravitreal anti-VEGF agent, KSI-301 (Kodiak Sciences, Palo Alto, CA). KSI-301 is an antibody biopolymer conjugate, meaning a novel full-length antibody against all forms of VEGF-A is covalently linked to an optically clear, branched, high molecular weight phosphorylcholine biopolymer via a single site-specific stable linkage. This approach leverages a larger size (950 kDa) with a 5 mg clinical dose in attempt to achieve extended intraocular duration of action. In comparison, aflibercept (Regeneron Pharmaceuticals, Tarrytown, NY) has a molecular weight of 115 kDa and a clinical dose of 2 mg. Preclinical modeling suggests that KSI-301 may be able to achieve an intraocular concentration at 3 months that is 1000 times greater than that of aflibercept. 130 patients (wAMD, n = 50; DME, n = 35; and RVO, n = 35) were randomized to 2 doses of KSI-301 and after 3 monthly loading doses, eyes were monitored monthly with protocol guided retreatment [16]. Impressively, among the wAMD arm 55% of eyes were reported to achieve a 6-month interval before a mandated first retreatment with 84% of eyes going 4 months or longer without retreatment. At 24 weeks (with a mean of 0.16 injections between weeks 8 and 24), BCVA had improved 5.9 letters compared to baseline. In the DME arm, 64% of eyes reached 6 months or longer without retreatment. In the RVO arm, 53% reached 4 months or longer without retreatment. No intraocular inflammation or ocular SAEs were reported in study eyes to date. A Phase 2, pivotal nAMD trial randomizing patients to aflibercept vs KSI-301 is underway [17].

Dr. Barry Kupperman reported results related to abicipar pegol for the treatment of nAMD (Allergan). Abicipar was engineered using DARPin technology in attempt to generate a novel anti-VEGF agent with high binding affinity. In the phase 3 CEDAR and SEQUOIA trials [18], the proportion of eyes with stable vision, losing less than 15 letters, was reported to be similar between eyes treated with abicipar every 8 weeks, abicipar every 12 weeks, and ranibizumab (Genentech) every 4 weeks through both 1 and 2 years of dosing. The biggest challenge appears to be inflammation. In the phase 3 program, the overall incidence of intraocular treatment emergent adverse events was about 15% in the abicipar arms compared 0.3% in the ranibizumab arm from baseline through week 52. Concerningly, a meaningful proportion of patients treated with abicipar who developed intraocular inflammatory events developing occlusive vasculitis. In a subsequent prospective trial, MAPLE, which used abicipar manufactured using a modified process, the overall incidence of inflammation was reported to decrease to 8.9% through 28 weeks of treatment and most events were reported to be mild to moderate in severity.

Combination therapies targeting angiopoietin-2 (ANG2) or VEGF-C and VEGF-D in addition to VEGF-A may allow improvement in visual and anatomic outcomes beyond VEGF-A monotherapies. Dr. Karl Csaky provided an update on clinical studies employing faricimab (Genentech) which is the first bispecific antibody designed for intraocular use [19], capable of simultaneously inhibiting both VEGF-A and angiopoietin 2 (ANG2). Faricimab has demonstrated sustained inhibition of both VEGF-A and ANG2 in human studies with evidence of increased durability beyond the effect of ranibizumab monotherapy in both DME [20] and nAMD phase 2 studies. The Phase 2 Stairway Trial demonstrated the potential for faricimab q 16-week dosing for the management of nAMD; 3 arms were studied: ranibizumab 0.5 mg every 4 weeks; faricimab 6 mg every 12 weeks and faricimab 6 mg every 16 weeks. BCVA at 52 weeks was equivalent for all 3 arms with 65% of faricimab eyes demonstrating no prespecified evidence of disease activity at week 24, 12 weeks after the last faricimab loading dose. Phase 3 trial programs in both nAMD and DME have been fully enrolled with anticipated data within the next year for the nAMD trials [20, 21].

Dr. Pravin Dugel presented the results of a Phase II clinical trial studying the use of OPT-302 (Opthea, South Yarra, Victoria, Australia) in combination with ranibizumab for the treatment of nAMD. OPT-302 (sVEGRr) is a “trap” inhibitor of VEGF-C and VEGF-D, which typically signal through VEGFR-2 and VEGFR-3. Currently used anti-VEGF agents (ranibizumab, bevacizumab (Genentech), aflibercept, and brolucizumab (Novartis, Basel, Switzerland) inhibit VEGF-A or VEGF-A, B and placental growth factor (aflibercept). This study hypothesized that pan-VEGF blockade would improve clinical outcomes. The trial included three treatment arms: 2.0 mg OPT-302 plus 0.5 mg ranibizumab; 0.5 mg OPT-302 plus 0.5 ranibizumab; 0.5 ranibizumab plus sham. All 3 arms received 6 monthly doses. Patients in the 2.0 mg OPT-302 plus 0.5 mg ranibizumab group gained a mean of 14.2 letters of vision from baseline compared to 10.8 letters in the ranibizumab monotherapy group (p = 0.0107) [22]. Progression into a global phase 3 program was discussed.

In addition to the development of new pharmaceutical agents that inhibit VEGF-A and other cytokines as described above, there are also promising drives to improve delivery of our current pharmaceutical agents. The port delivery system (PDS, Genentech) utilizes a hardware approach to sustained delivery of ranibizumab. Gene therapy is also being pursued in which viral vectors are used to create an intraocular bio-factory for the production of either aflibercept or ranibizumab.

Dr. Carl Regillo presented end of study results of the Ladder phase II trial which studied the PDS in nAMD management [23]. The PDS is a transscleral device which is surgically implanted at the pars plana that serves are a refillable intraocular reservoir allowing continuous delivery of ranibizumab by diffusion. The PDS can be refilled in clinic using a proprietary flushing-syringe by accessing the PDS through the overlying conjunctiva and Tenon’s layer. In the PDS 100 mg/ml arm of Ladder, approximately 80% of patients went ≥ 6 months and approximately 60% went ≥ 12 months without meeting pre-defined specific refill criteria. In patients who received ≥ 1 refill, median time to first and second refills was consistent at about 8.8 months. Top-line serum pharmacokinetic data appeared to support that the PDS implant on average is continuously delivering ranibizumab through at least 12 months. A Phase III trial involving nAMD [24] has completed enrollment with data anticipated in 2020 and phase 3 trials in both DME and DR are underway [25].

Two presentations described data related to gene therapy approaches to nAMD. Dr Jeffery Heier described an approach using an adeno-associated virus (AAV)-8 vector delivered by sub-retinal injection during a pars plana vitrectomy carrying a gene encoding a molecule similar to ranibizumab, an anti-VEGF fragment antigen-binding (Regenxbio, Rockville, MD) [26]. Dr David Boyer described an approach using an AAV.7m8 vector delivered by intravitreal injection carrying a gene encoding a molecule similar to aflibercept (Adverum Biotechnologies, Menlo Park, CA) [27]. Both approaches continue to show promising results with reliable gene expression and protein production in animal models and early phase human studies. Discussion of the possibility of moving into phase 2 programs in both nAMD and DR with both agents was discussed.

While treatments of active nAMD advance, another approach to optimizing outcomes could be to slow conversation of intermediate dry AMD to the neovascular form. Towards this end, Dr. Jeffrey Heier presented 2-year results of the investigator-initiated ProCon study which investigated the effectiveness of quarterly aflibercept dosing for preventing conversion of high-risk eyes with dry AMD to nAMD [28]. Treatment was performed over 2 years. High risk was defined as intermediate dry AMD in one eye and a history of nAMD in the fellow eye. Aflibercept quarterly prophylaxis did not affect conversion rates through month 24. At 24 months, the rate of conversion to nAMD was 9.52% in the treated group (n = 63) and 10.9% (n = 64) in the sham group. A history of nAMD diagnosis in a fellow eye ≤ 2 years prior to enrollment in ProCon was associated with a higher nAMD conversion rate. In eyes with non-exudative CNV as demonstrated by OCT angiography (OCT-A) at baseline, the rates of nAMD conversion were meaningfully higher (27% in the treated group and 31% in the sham group). Development or progression of GA was not affected by quarterly aflibercept treatment. The conclusion of this 2-year study supports the current management consensus that anti-VEGF dosing be initiated after exudation develops, with the consideration that once exudation manifests, earlier treatment leads to better absolute outcomes [29].

Dr. Anat Loewenstein presented updates on the development of a low-cost, AI-enabled, patient self-operated home OCT system (Notal Vision, Manassas, VA). It had been previously reported that patients could successfully self-image with the Home OCT prototype device, and graders could identify retinal fluid with 98% sensitivity and 97% specificity in these Home OCT images when compared to images taken with a commercial device. This year, the final device configuration was shown, with examples of image quality for fluid identification which were consistent with the Spectralis device. A deep learning algorithm, the Notal OCT Analyzer (NOA) was used to identify minute quantities of intra and sub-retinal fluid, on the nano-liter scale, which were also represented in fluid thickness maps. A longitudinal analysis was performed on a patient, and the algorithm was able to detect amounts as small as 18 pico-liters. The patient self-testing with the Home OCT demonstrated the longitudinal disease dynamics, with the detection of a 7× increased amount of fluid within 4 days to 124 nl, and subsequent resolution of 3x within 3 days followed by continuous resolution after treatment with anti-VEGF therapy. The information generated by tele-connected OCT in patients’ homes has the potential to support current and future advances and retinal disease management.

## Drug delivery and devices

Suprachoroidal approaches continue to be investigated as a route for therapeutics targeting retinal diseases. Dr. Thomas Albini reviewed the use of a proprietary microinjector (Clearside Biomedical, Alpharetta, Georgia, USA) for precise delivery into the suprachoroidal space (SCS). There are multiple theoretical advantages with suprachoroidal delivery: (a) targeting the therapeutic effect to the retina and choroid while minimizing exposure to anterior segment structures such as the filtration angle and crystalline lens and (b) no entry into the vitreous cavity is required. The Phase 3 Peachtree trial [30] demonstrated the safety and efficacy of this system for delivering triamcinolone acetate (TA) for the treatment of cystoid macular edema secondary to uveitis. In contrast to what would be expected with intravitreal delivery of TA, there was a lower signal of intraocular pressure increase and cataract development following suprachoroidal delivery. Suprachoroidal delivery of a gene therapy has also been reported to achieve similar expression of an anti-VEGF Fab as achieved with subretinal delivery with corresponding functional suppression of vascular leakage [31].

## Diabetic retinopathy

Dr. Charles Wykoff presented 2-year results from the phase III, double-masked PANORAMA trial that randomized treatment-naïve eyes with moderately severe to severe NPDR without center-involved DME (n = 402) to either sham or 2 dosing regimens of aflibercept. In the 2nd year, the every 4-month (Q16) aflibercept arm continued fixed Q16 dosing and maintained the proportion of patients who achieved an improvement of two or more diabetic retinopathy severity scale (DRSS) steps at about 62% with a mean of just 2.6 injections. In comparison, in the 2nd year the every 2-month (Q8) aflibercept arm transitioned to PRN re-treatment based on investigator determined DRSS level; using this approach, the proportion of patients who achieved an improvement of two or more DRSS steps decreased from 80% at 1 year to 50% at 2 years. Of primary clinical relevance, using a Kaplan–Meier analysis accounting for discontinued patients, nearly 58% of sham eyes developed either PDR or CI-DME by the end of year 2, a proportion reduced by about 75% with aflibercept dosing using either approach to about 19% [32].

Dr. Harry Flynn described the use of wide-field (WF) OCT-A in the diagnosis and follow-up care of eyes with proliferative DR (PDR). OCT-A was found to be as effective as, or even more effective than, WF fluorescein angiography (FA) for identifying and following both areas of neovascularization as well as areas of retinal non-perfusion in and near the posterior pole. The added value of WF-FA imaging was the inclusion of more far-peripheral pathology than was captured using OCT-A along. High speed SS-OCT coupled with software montage techniques provided the highest quality WF images. Future clinical studies involving DR should consider using both SS-OCT-A and WF-FA as relevant clinical endpoints.

Dr. Michael Ip discussed utilization of AI for DR screening and presented the results for the EyeArt (Eyenuk, Inc, Woodland Hills, CA, USA) multicenter prospective clinical trial. Current approach to screening patients with diabetes mellitus for DR in the United States are not achieving adequate penetration. This study assessed the sensitivity and specificity of the EyeArt system in detecting referable DR and vision threatening DR by analyzing 2-field non-mydriatic fundus photographs. The clinical reference was standardized, adjudicated DR grading performed by the Wisconsin Fundus Photograph Reading Center. The EyeArt system, with no pupillary dilation, achieved a remarkable sensitivity of 95.5%, less impressive specificity at 86.0% and gradeability of 87.5%. This study demonstrated that the EyeArt system may be able to be used to improve rates of DR screening and may be valuable in assisting our health care system at identify and triaging patients requiring ophthalmology evaluation and possible intervention for this largely preventable cause of blindness.

## Conclusions

There remains a tremendous amount of activity in the space of research targeting the development and refinement of therapeutics for retinal diseases. The 2020 *Angiogenesis, Exudation and Degeneration Symposium* highlighted some of the most promising innovations shaping the landscape of clinical research in retinal diseases. Both the intermediate and advanced forms of dry AMD remain an enormous un-met need, and multiple therapeutics including elamipretide, risuteganib, pegcetacoplan, avacincaptad, brimonidine and GR39821 are being explored for potential benefit in this arena. Novel pharmaceutical agents including KSI-301, abicipar, faricimab and OPT-302 are being investigated for their capacity to improve efficacy and durability beyond our current anti-VEGF monotherapies. Gene therapy approaches in ADVM-022 and RGX-314 as well as surgical devices such as the PDS and are being pursued for their promise of delivering more durable anti-VEGF activity. Woven into these development programs and promising to improve prognostication are improved imaging systems and validated AI algorithms.

## Data Availability

Not applicable.
